# Effects of Glucose Fluctuation Targeted Intervention on the Prognosis of Patients with Type 2 Diabetes following the First Episode of Cerebral Infarction

**DOI:** 10.1155/2020/2532171

**Published:** 2020-01-28

**Authors:** Qingqing Lou, Xiaodan Yuan, Shujie Hao, Joshua D. Miller, Juan Yan, Panpan Zuo, Jianing Li, Lihong Yang, Hong Li

**Affiliations:** ^1^Sir Run Run Shaw Hospital, Zhejiang University School of Medicine, Department of Endocrinology, Hangzhou 310016, China; ^2^Affiliated Hospital of Integrated Traditional Chinese and Western Medicine, Nanjing University of Chinese Medicine, Department of Health Education, Nanjing 210028, China; ^3^Anhui University of Chinese Medicine, Nursing College, Hefei 230012, China; ^4^Renaissance School of Medicine at Stony Brook, Department of Medicine, NY 11794-8154, USA; ^5^Nanjing University of Chinese Medicine, Nursing College, Nanjing 210023, China; ^6^The Affiliated Suzhou Hospital of Nanjing Medical University, China

## Abstract

**Objective:**

The purpose of this study was to assess the effects of glucose fluctuation targeted intervention on neurologic function, independent living skills, and quality of life in type 2 diabetes patients following the first episode of cerebral infarction (CI).

**Methods:**

This was a randomized control trial. Following confirmed cerebral infarction, 75 patients with type 2 diabetes were randomized into 2 groups: control group (*n* = 37) with usual care, focused on hemoglobin A1c (HbA1c) control, targeting A1c < 7%, and intervention group (*n* = 37) with usual care, focused on hemoglobin A1c (HbA1c) control, targeting A1c < 7%, and intervention group (

**Results:**

After 6 months, data from 63 patients were analyzed (30 in the control group, 33 in the intervention group). There was no difference (*P* > 0.05) in the reduction of A1c between the 2 groups, but the reductions of LAGE (*P* > 0.05) in the reduction of A1c between the 2 groups, but the reductions of LAGE (*P* > 0.05) in the reduction of A1c between the 2 groups, but the reductions of LAGE (*P* > 0.05) in the reduction of A1c between the 2 groups, but the reductions of LAGE (*P* > 0.05) in the reduction of A1c between the 2 groups, but the reductions of LAGE (*P* > 0.05) in the reduction of A1c between the 2 groups, but the reductions of LAGE (*P* > 0.05) in the reduction of A1c between the 2 groups, but the reductions of LAGE (*P* > 0.05) in the reduction of A1c between the 2 groups, but the reductions of LAGE (*P* > 0.05) in the reduction of A1c between the 2 groups, but the reductions of LAGE (

**Conclusion:**

Glucose fluctuation targeted intervention can improve nerve function for patients with T2DM following the first CI episode. This trial is registered with NCT03932084.

## 1. Introduction

Diabetes mellitus is an independent risk factor for cerebral infarction. People with diabetes have more than double the risk for cerebrovascular diseases, after controlling for confounding, relative to individuals without the disease [1]. Interestingly, even in patients without known diabetes, hyperglycemia occurs in 20-40% following acute ischemic stroke [2].

The relationship between HbA1c and risk of microvascular disease in patients with diabetes is well-established; the impact of tighter glycemic control on macrovascular complications, however, is not as robust [3–5]. Since HbA1c neither captures glycemic variation nor does it provide any information on glucose dynamics [6], we propose that the use of HbA1c alone is not sufficient to assess the risk for macrovascular complications and the metabolic status of patients with type 2 diabetes mellitus (T2DM).

Glucose fluctuation, also known as glucose variability (GV), refers to variations in blood glucose levels and, more broadly, to blood glucose oscillations that occur throughout the day. These may include periods of hypoglycemia and postprandial hyperglycemia, as well as day-to-day glycemic variations [7]. Recent studies have demonstrated that compared with sustained hyperglycemia, increased glycemic variation has been considered a major risk factor in the development of diabetes macrovascular complications [8–10]. Utilizing a continuous glucose monitoring system (CGMS), an Italian observational study with a cohort of 1,409 subjects with T2DM aged 56–74 years followed up to 10 years found that fasting plasma glucose variation coefficient (CV-FPG) was an independent predictor of cardiovascular mortality [11], even in patients with similar mean glucose levels [12]. A recent study [13] revealed that patients with elevated glycemic variability had significantly higher SYNTAX scores, an indicator of coronary artery lesion severity. Indeed, SYNTAX scores remained significantly associated with high GV, independent of HbA1c. There is a significant association between glycemic fluctuation and the incidence of diabetes macrovascular complications. Therefore, understanding the impact of glycemic fluctuations on diabetic macroangiopathy could be inform strategy for prevention and treatment of macroangiopathy in patients with diabetes.

Previous studies have been observational. To our knowledge, however, there has been no prospective intervention study confirming the effects of glucose fluctuation targeted management on the prognosis of T2DM patients following CI. The present randomized control trial sought to establish glucose fluctuation as an important target for management on metabolic index, neurologic function, and quality of life in T2DM patients following CI.

## 2. Methods

### 2.1. Participants

Participants were recruited between February 2017 and July 2017 from the Departments of Neurology and Endocrinology at the Hospital on Integration of Chinese and Western Medicine, an affiliate of the Nanjing University of Chinese Medicine. One hundred and four participants with a history of T2DM following CI were recruited. All participants underwent a medical examination, and a total of eighty-four participants fulfilled the inclusion criteria. Finally, 75 participants were willing to participate in this project.

### 2.2. Inclusion and Exclusion Criteria

The inclusion criteria were subjects (1) aged ≥18 years; (2) with CI within one month, diagnosed by magnetic resonance imaging (MRI) or computed tomography (CT) according to 1995 acute cerebral infarction diagnosis standards promulgated by the Fourth National Cerebrovascular Disease Conference [14]; and (3) having T2DM (as defined by the WHO diagnostic criteria in 1999) [15]. Exclusion criteria were (1) coexisting acute complications of diabetes including diabetic ketoacidosis (DKA), hyperglycemic hyperosmolar syndrome (HHS), and metabolic acidosis; (2) severe comorbid chronic complications of diabetes; (3) active malignancy; (4) subjects with mental illness and communication disorders; and (5) those actively participating in other research studies.

### 2.3. Sample Size

Sample size was calculated considering the expected change of the NIHSS score of the participants. NIHSS score was the primary study endpoint; our previous preliminary experiment has reported a reduction of the NIHSS score by 2.18. Sample size was calculated to have 90% power to detect a moderate 2.0-SD difference in the NIHSS score, with an alpha/*α* value of 0.05. Additional participants were recruited until the required number was achieved (*n* = 23) per group, with an anticipated dropout rate of 20%. Thus, we planned to recruit 66 subjects. Among 84 eligible participants, 75 subjects finally participated in this study.

### 2.4. Trial Design and Randomization

**Figure 1:**
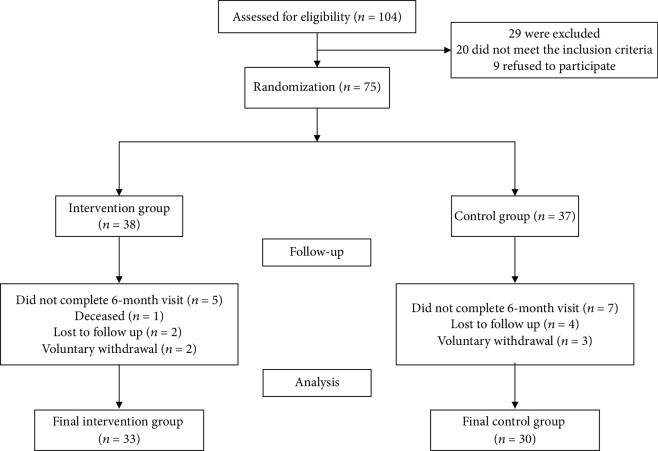
Flow diagram of study enrollment.

A total of 75 participants met inclusion criteria and were willing to participate in the study. Participants were randomized into two groups: (1) control group (*n* = 37) and (2) intervention group (*n* = 38) (Figure 1). The study was approved by the ethics committee of the Affiliated Hospital of Integrated Traditional Chinese and Western Medicine. Participants provided written informed consent prior to enrollment. All methods were carried out in accordance with approved guidelines and regulations.

### 2.5. Scales

#### 2.5.1. National Institutes of Health Stroke Scale (NIHSS)

The NIHSS is an impairment scale used to measure stroke severity. It was originally developed in 1989 [16] and is now widely used in clinics and is recommended as a valid tool to assess stroke severity in emergency departments.

The NIHSS includes the following domains: level of consciousness, sensory, neglect, visual field, gaze, facial palsy, motor arm, motor leg, limb ataxia, language, and dysarthria. Each domain is scored on an ordinal scale ranging from 0 to 2, 0 to 3, or 0 to 4. Item scores are summed to a total score ranging from 0 to 42 (the higher the score, the more severe the stroke).

#### 2.5.2. Modified Rankin Scale (MRS)

Stroke outcome is most commonly rated by the modified Rankin scale [17] because of its well-established validity and rapid application; it can discriminate clinically relevant levels of disability and recovery in clinical trials [18–20]. We used a modified Rankin scale [21] to create a more comprehensive assessment of CI patients' independence and living ability to also include physical function and activities of daily living (ADLs). The 0-6 Likert scale is as follows: 0, no symptoms; 1, no significant disability; 2, slight disability; 3, moderate disability; 4, moderately severe disability; 5, severe disability; and 6, dead. The higher the score, the worse the patient's prognosis. When evaluating prognosis, a score≦2 was classified as a “good”.

#### 2.5.3. Stroke Impact Scale (SIS)

The Stroke Impact Scale is a specific scale that evaluates disability and health-related quality of life after stroke [22]. The scale has been translated into Chinese, with well-validated reliability and sensitivity [23]. The overall Cronbach coefficient is 0.923, and the test-retest reliability is 0.712-0.912, accurately reflecting quality of life in patients with CI as well as and potential change over time. The SIS includes the following eight domains: strength, memory, thinking, emotion, communication and ADLs, mobility, hand function, and participation. A total of 8 domains with 59 items are scored on a 5-point scale, of which the sixth, eighth, and ninth questions of the emotional dimension are reversed, and the other items are positive. Each domain scores range from 0 to 100 and are calculated using the following equation: calculated score = [(actual score − the lowest possible score in this domain)/(the difference between the highest possible score and the lowest score in this domain)] × 100. The total score of the SIS scale is the sum of all domains. A higher score indicates better quality of life and less functional damage.

### 2.6. Study Procedure

#### 2.6.1. Control Group

The control group received usual care as follows:

*(1) During Hospitalization*.

1. Monitor subjects' blood glucose by point-of-care capillary blood glucose testing (Yueyou II, Yuwell) during hospitalization (fasting, two-hour postprandial, and bedtime) and calculate as the difference between the highest and the lowest blood glucose values during one day (LAGE), the ratio of standard deviation and mean (CV-FPG)
2. Individual diabetes education is the one-on-one education for patients and their families by diabetes specialist nurses. Educational content takes 60 minutes to deliver and includes skills related to diabetes self-management, basic knowledge of diabetes, diet, exercise, medication, blood glucose monitoring, risks of glucose fluctuations, and hypoglycemia. Insulin self-administration skills were taught and reinforced to those patients requiring insulin as part of their management plan
3. Teaching patients and their families to use blood glucose meters and correctly record results. The diabetes specialist nurses demonstrate correct methods for self-monitoring blood glucose, explain the operation precautions, and ask the patient or family to perform repeated operation training under supervision until skills are mastered

*(2) During Discharge*. Patients were given standard hospital discharge instructions, home blood glucose monitors, test strips, and log books and were asked to monitor their blood glucose 5 times (fasting, 2 hours postprandially, and before sleep) daily following hospital discharge.

*(3) Follow-Up*. Within-range blood sugars did not prompt intervention (fasting plasma glucose (FPG) < 7 mmol/L(126 mg/dL), 2hPG < 10 mmol/L (180 mg/dL), and A1c < 7%, no hypoglycemia). If hyper- or hypoglycemia did occur, researchers (diabetes educators) explored potential causes with each patient. Causes related to lifestyle (such as dietary indiscretion and unanticipated exertion) prompted exploration of self-care behavioral solutions between the patient and the educator. For an absent obvious cause, physician endocrinology referral was made for potential medication adjustment and further evaluation. Participants received telephone follow-up one week following discharge; thereafter, follow-up was conducted monthly for six months.

#### 2.6.2. Intervention Group

**Figure 2:**
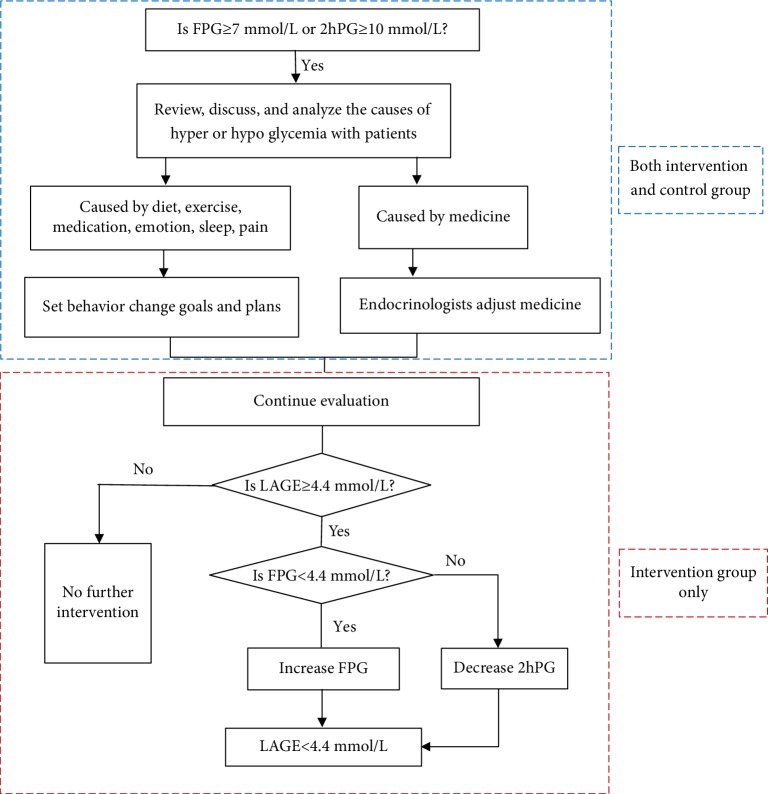
Flow diagram for two groups.

In addition to control group intervention parameters, we also added LAGE < 80 mg/dL as one management goal. Even if FBG, 2hPG, and A1c were all within the target range, LAGE ≥ 80 mg/dL prompted initial careful assessment of dietary intake, physical activity, and activities of daily living. If out-of-range LAGE was attributed to lifestyle, researchers would work with patients to find a self-care behavioral solution for the glycemic fluctuation and set behavioral goals. For an absent apparent cause for abnormal LAGE, patients were referred to a physician endocrinologist for further guidance. If a patient's FPG was <80 mg/dL, the physician would adjust the medications for the patient to increase his or her FPG to minify the LAGE; otherwise, the doctor would increase the patient's dosage or medications to decrease the 2hPG. At subsequent follow-up visit, we evaluated glucose fluctuation and target completion.  Figure 2 details follow-up visit procedures. As an example, if a patient's FPG was 5 mmol/L (90 mg/dL), and his 2hPG was 9.5 mmol/L (171 mg/dL), he would receive no intervention if he was in the control group, but if he was in the intervention group, the team would aim to reduce his 2hPG, thus targeting a LAGE < 80 mg/dL.

### 2.7. Data Analysis

All analyses were performed using IBM SPSS version 22 (IBM Corp, Armonk, NY, USA). Data was presented as mean ± SD; the significance of differences in outcomes between the two groups was assessed using *t*-tests when the data was normally distributed and using nonparametric tests of two independent samples when the data was not normally distributed. Within-group differences were tested by the Wilcoxon signed-rank or paired *t*-test. Categorical variables were tested by chi-square test. Statistical significance was established at the *P* < 0.05 level.

## 3. Results

A total of 75 participants were enrolled in the study and were randomly assigned to the intervention group (*n* = 38) or the control group (*n* = 37). By study's end, a total of 63 participants completed the 6-month intervention (16% dropout rate). The reasons for dropping out were the voluntary withdrawal and the loss to follow-up (*n* = 5, *n* = 6), and one participant was deceased (Figure 1).

**Figure 3:**
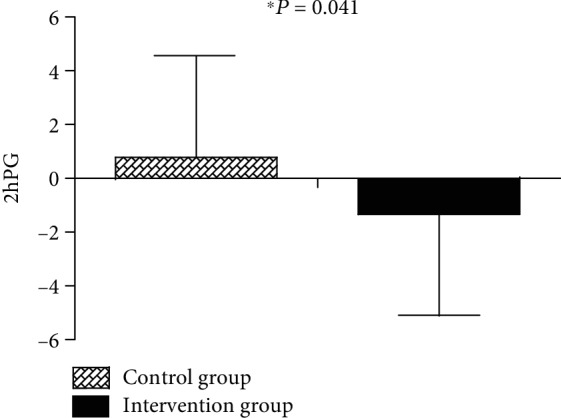
Changes in 2hPG from baseline to 6 months between the control and glucose fluctuation target management groups.

**Figure 4:**
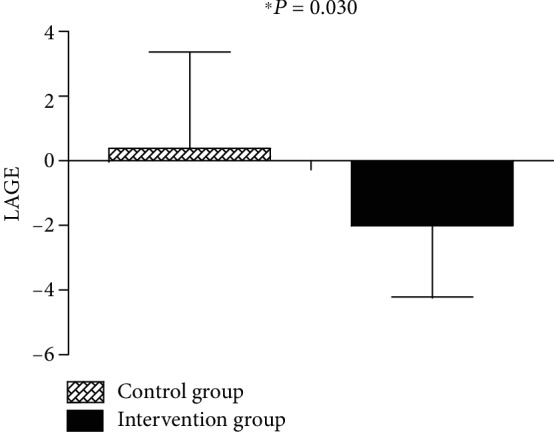
Changes in LAGE from baseline to 6 months between the control and glucose fluctuation target management groups.

**Figure 5:**
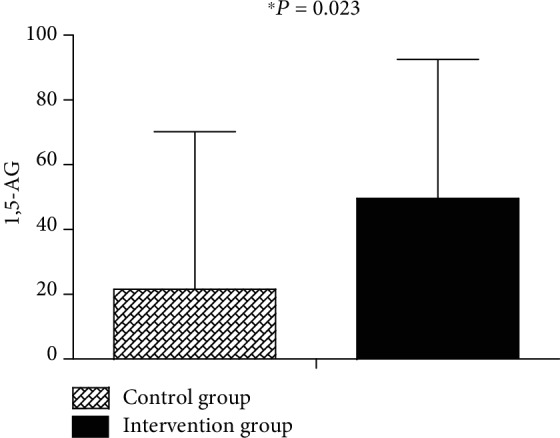
Changes in 1,5-AG from baseline to 6 months between the control and glucose fluctuation target management groups.

As shown in Table 1, the two groups did not differ significantly at baseline. Following intervention, there was no difference in HbA1c between the intervention group and the control group (*P* > 0.05) (Table 2); the difference values of LDL-c (*P* = 0.046), 2hPG (*P* = 0.041), and LAGE (*P* = 0.030) were significantly decreased, whereas the 1,5-AG (*P* = 0.023) level was significantly increased in the intervention group compared to the control group (Figures 3–5).

Within the intervention group, 2hPG (*P* = 0.041), HbA1c (*P* = 0.011), and LAGE (*P* = 0.015) were significantly reduced, whereas the 1,5-AG was significantly improved (*P* < 0.001) after 6 months (Table 3).

Within the control group, hip circumference (HC), high-density lipoprotein cholesterol (HDL-c), LDL-c, and 1,5-AG were improved compared to baseline (*P* < 0.001) (Table 4).

There was no difference in the NIHSS score between the groups (*P* > 0.05) at baseline; after 6 months, the NIHSS score was significantly lower in the intervention group compared to the control group (*P* = 0.012) (Table 5).

There was no significant difference in the scores of each dimension and total scores of SIS between the two groups before intervention (*P* > 0.05). After 6 months, the control group had a total score of 559.43 ± 112.53, and the intervention group had a total score of 615.47 ± 87.94. There was no statistically significant difference between the two groups. However, in the two dimensions of strength and hand function, the intervention group had a statistically significant better performance (*P* < 0.05) (Table 6).

The changes in the blood glucose measurement value at the 5 points (blood glucose self-monitoring by finger stick) and hypoglycemia were reported in Table 7. There was no difference between two groups in the changes of 5 points of blood glucose measurement and hypoglycemia occurrence.

## 4. Discussion

This is the first prospective study examining the impact of glucose fluctuation targeted intervention on outcomes in patients suffering a neurological insult. We found that after 6-month individualized glucose fluctuation target management, nonsignificant differences were observed in HbA1c between groups. Interestingly, our data noted a statistical improvement in LAGE, 1,5-AG, nerve function, and quality of life in the intervention group compared with the control group.

In the current study, due to the synergistic effects of behavioral intervention and drug regimen adjustment, LAGE, an index reflecting intraday blood glucose fluctuations, decreased significantly in the intervention group. Because of nonsignificant differences in HbA1c at baseline, we attributed the benefits in LAGE to glucose fluctuation target management. Our study suggested that it is worth paying additional attention to the LAGE (≥4.4 mmol/L) and making corrections by means of lifestyle intervention or medication adjustment.

After 6-month individualized glucose fluctuation target management, 1,5-AG increased significantly in the intervention group when compared with controls. 1,5-AG is a very sensitive and reliable clinical index. It has proven useful in predicting short-term blood glucose fluctuation (within 1-2 weeks), HbA1c is normal or only slightly elevated and can adequately reflect postprandial hyperglycemia and blood glucose fluctuation [24]. 1,5-AG can predict CD8+T and CD4+T cell modifications related to atherosclerosis [25]. A Japanese study verified that decreased levels of 1,5-AG were a risk factor for macrovascular diseases, especially in males [26]. In addition, patients with low levels of 1,5-AG were 4.07 times more likely to have CI than those with high levels of 1,5-AG [27]. In our study, we included patients with CI. Therefore, whether increased 1,5-AG is beneficial to prevent recurrence of CI remains to be studied.

In our study, the intervention group demonstrated decreased neurologic impairment when compared to controls. It is worth noting that there was no statistically significant difference between the two groups in HbA1c following intervention, but LAGE and 1,5-AG were significantly improved in the intervention group. This indicates that improvement in neurologic function in the intervention group may have been due to reduction in glucose fluctuation. Long-term data from UKPDS, DCCT, VADT, and other large studies have confirmed that controlling HbA1c to the ideal level can significantly reduce the occurrence of microvascular complications but cannot necessarily effectively reduce the incidence of cardiovascular and cerebrovascular events in patients with diabetes [28].

Our findings conclude that following 6-month glucose fluctuation target management, the MRS score, used as the evaluation tool for predicting CI prognosis [18], was lower in the intervention group (1.48 ± 0.76) than the control group (1.53 ± 0.90). Furthermore, 90.91% of patients in the intervention group had a good prognosis (MRS ≤ 2) with only 86.67% in the control group. Nevertheless, we did not observe statistically significant between-group differences in the MRS scores. Two reasons might account for this difference: first, the present study had a relatively small sample size and short intervention duration; and, second, subjects included in this study were motivated patients with mild disease. Previous data showed that amplitude of glucose fluctuation may directly affect or even determine the prognosis of T2DM patients with CI [29], indicating that glucose fluctuation target management has an important effect on the prognosis of vascular complications of diabetes. Therefore, in a future study, we could extend the intervention duration and include patients with more severe disease to verify the effects of glucose fluctuation target management on patient prognosis.

Our finding revealed that after 6-month glucose fluctuation target management, there was no statistically significant difference in total scores of SIS in patients with glucose fluctuation target management when compared to usual care. However, in the intervention group, strength, hand function, and participation dimensions of SIS increased significantly relative to the control group. In this study, we found that patients with postprandial hyperglycemia benefitted from focusing on dietary guidance, postprandial exercise guidance by way of increased strength, and hand function. Furthermore, “one-to-one” follow-up encouraged patients to communicate with their clinical team, to investigate causes of dysglycemia and fluctuations in overall glycemic control and set behavioral change goals, greatly improving the patient's ability to participate in diabetes self-care. In contrast, Ma et al. [30] reported that blood glucose management in patients with T2DM combined with CI can improve ADLs. This discrepancy may be related to the fact that patients in their study received systematic rehabilitation early in their hospitalization. But in our study, we mainly focused on glucose fluctuation target management and provided some exercise guidance according to the patient's glycemic fluctuation value, for an absent global approach to systematic rehabilitation training. Thus, it should be noted that both the glucose fluctuation target management and the rehabilitation of nerve function are indispensable for patients with T2DM combined with CI. A future study to standardize the rehabilitation training of patients after discharge is needed.

At present, most studies on the glycemic fluctuation in T2DM patients with CI were cross-sectional studies. To our knowledge, ours is the first prospective study to target glucose *fluctuation* management. Glucose fluctuation target management can be generalized to T2DM patients with cerebrovascular disease in the further research.

Limitations of this study warrant mention. First, the overall sample size was relatively small and the subjects included in this study were patients with mild disease who were willing to cooperate, which may be biased and limit the power of some conclusions. Second, the 6-month intervention in this study was relatively short. Future studies involving the intervention time can be extended to 1-2 years. Third, due to budget limitations, instead of continuing glucose monitoring (CGM), self-monitoring of blood glucose (SMBG) was used to evaluate glycemic fluctuation, and blood glucose values before lunch and dinner were not routinely checked, which could increase the risk of hypoglycemia. In future studies, CGM will be used to assess glucose fluctuation for more comprehensive and accurate data.

In conclusion, the results of this study suggested that after 6 months, patients receiving glucose fluctuation management achieved better 2hPG, 1,5-AG, LAGE, and LDL-c, reduced neurologic defect, and improved the quality of life when compared to controls. However, both the control and intervention groups had similar effects in increasing functional status. This study provides valuable information to guide diabetes clinicians in targeting glucose *fluctuation* in order to promote better glycemic control, lipid profile, and overall quality of life in patients with DM-related macrovascular disease.

## Figures and Tables

**Table 1:** Demographic characteristics and clinical variables at baseline (*n* = 63).

Characteristics	Control *n* = 30	Intervention *n* = 33	*χ* ^2^	*P* value
Gender, *n* (%)			0.551	0.458
Male	20 (66.7%)	19 (57.6%)		
Female	10 (33.3%)	14 (42.4%)		
Family history of CI, *n* (%)			0.002	0.961
No	18 (60.0%)	20 (60.6%)		
Yes	12 (40.0%)	13 (39.4%)		
Family history of diabetes, *n* (%)			0.148	0.701
No	16 (53.3%)	16 (48.5%)		
Yes	14 (46.7%)	17 (51.5%)		
Marital status, *n* (%)			0.223	0.223
Not married	0 (0.0%)	0 (0.0%)		
Married	28 (93.3%)	33 (100%)		
Divorced/widowed	2 (6.7%)	0 (0.0%)		
Educational level, *n* (%)			5.418	0.228
Illiterate	0 (0.0%)	0 (0.0%)		
Primary school	4 (13.3%)	3 (9.1%)		
Middle school	16 (53.4%)	18 (54.6%)		
High school/secondary school	4 (13.3%)	10 (30.3%)		
Associate	5 (16.7%)	1 (3.0%)		
Bachelor and above	1 (3.3%)	1 (3.0%)		
Medical fee payment method, *n* (%)			0.007	0.933
Free medical care	0 (0.0%)	0 (0.0%)		
Self-payment	2 (6.7%)	1 (3.0%)		
National health insurance	28 (93.3%)	32 (97.0%)		
Career, *n* (%)			1.371	0.242
In-service	7 (23.3%)	4 (12.1%)		
Nonservice	23 (76.7%)	29 (87.9%)		
Comorbidities, *n* (%)			2.618	0.106
No	4 (13.3%)	10 (30.3%)		
Yes	26 (86.7%)	23 (69.7%)		
Diabetes complications, *n* (%)			1.801	0.18
No	15 (50.0%)	11 (33.3%)		
Yes	15 (50.0%)	22 (66.7%)		
Injecting insulin, *n* (%)			0.716	0.397
No	15 (50.0%)	13 (39.4%)		
Yes	15 (50.0%)	20 (60.6%)		
Smoking, *n* (%)			0.682	0.409
No	23 (76.7%)	28 (84.8%)		
Yes	7 (23.3%)	5 (15.2%)		
Drinking, *n* (%)			3.067	0.08
No	21 (70.0%)	29 (87.9%)		
Yes	9 (30%)	4 (12.1%)		
Exercise, *n* (%)			0.05	0.824
No	11 (36.7%)	13 (39.4%)		
Yes	19 (63.3%)	20 (60.6%)		
Age (years)	65.37 ± 9.01	64.03 ± 6.93	0.663	0.51
Weight (kg)	67.81 ± 11.94	66.28 ± 10.29	0.546	0.587
BMI (kg/m^2^)	25.05 ± 3.37	25.03 ± 3.26	0.015	0.988
WC (cm)	89.00 ± 10.21	87.67 ± 10.37	0.513	0.61
HC (cm)	94.85 ± 7.03	96.14 ± 5.94	-0.787	0.435
SBP (mmHg)	148.10 ± 20.41	144.27 ± 20.53	0.741	0.461
DBP (mmHg)	85.10 ± 10.20	82.42 ± 20.19	0.654	0.516
TC (mmol/L)	4.32 ± 1.02	4.20 ± 1.39	0.38	0.705
TG (mmol/L)	1.83 ± 1.16	1.36 ± 0.90	1.874	0.061
HDL-c (mmol/L)	1.21 ± 0.39	1.35 ± 0.54	1.572	0.116
LDL-c (mmol/L)	2.62 ± 0.97	2.63 ± 1.11	-0.026	0.98
FPG (mmol/L)	8.92 ± 3.27	7.54 ± 2.72	1.809	0.076
2hPG (mmol/L)	12.40 ± 3.58	10.66 ± 3.66	1.894	0.063
HbA1c (%)	8.15 ± 2.46	7.91 ± 2.16	0.404	0.688
1,5-AG (*μ*g/mL)	90.14 ± 36.69	97.81 ± 34.85	-0.841	0.402
CV-FPG (%)	0.20 ± 0.12	0.19 ± 0.11	0.277	0.783
LAGE (mmol/L)	5.96 ± 2.16	5.84 ± 2.21	0.202	0.841

The data are shown as *n* (%) and mean ± SD. CI: cerebral infarction; BMI: body mass index; WC: waist circumference; HC: hip circumference; SBP: systolic blood pressure; DBP: diastolic blood pressure; TC: total cholesterol; TG: triglyceride; HDL-c: high-density lipoprotein cholesterol; LDL-c: low-density lipoprotein cholesterol; FPG: fasting plasma glucose; 2hPG: 2-hour postprandial blood glucose; HbA1c: hemoglobin A1c; 1,5-AG: 1,5-anhydroglucitol; CV-FPG: fasting plasma glucose variation coefficient; LAGE: largest amplitude of glycemic excursions.

**Table 2:** Before and after glucose fluctuation target management between-group comparison of changes on glycemic control and lipid profile variables (postintervention baseline).

Variables	Control *n* = 30	Intervention *n* = 33	*T*/*Z*	*P* value
Weight (kg)	0.97 ± 2.47	0.94 ± 4.00	-0.031	0.975
BMI (kg/m^2^)	0.38 ± 0.91	0.40 ± 1.52	0.079	0.938
WC (cm)	1.80 ± 6.59	0.70 ± 6.33	-0.639	0.525
HC (cm)	3.76 ± 6.55	2.41 ± 6.69	-0.765	0.447
SBP (mmHg)	4.52 ± 17.40	1.58 ± 18.13	-0.6	0.551
DBP (mmHg)	1.83 ± 12.67	1.71 ± 17.16	-0.027	0.978
TC (mmol/L)	0.26 ± 0.64	−0.06 ± 1.06	-1.331	0.189
TG (mmol/L)	−0.31 ± 1.11	0.17 ± 0.99	1.686	0.098
HDL-c (mmol/L)	0.17 ± 0.23	−0.12 ± 0.46	-1.805	0.077
LDL-c (mmol/L)	0.25 ± 0.57	−0.12 ± 0.77	-2.043	0.046^∗^
FPG (mmol/L)	−0.89 ± 2.60	−0.34 ± 3.03	0.734	0.466
2hPG (mmol/L)	0.79 ± 3.70	−1.35 ± 3.65	-2.092	0.041^∗^
HbA1c (%)	−0.61 ± 1.75	−0.65 ± 1.33	0.143	0.887
1,5-AG (*μ*g/mL)	21.64 ± 47.81	49.65 ± 42.10	-2.342	0.023^∗^
CV-FPG (%)	−0.05 ± 0.31	−0.01 ± 0.15	0.916	0.378
LAGE (mmol/L)	0.38 ± 2.91	−2.01 ± 2.13	2.324	0.030^∗^

Data are shown as mean ± SD. Statistically significant figures are indicated by symbols, ^∗^*P* < 0.05.

**Table 3:** Comparisons of glycemic control and lipid profile variables within the glucose fluctuation target management group.

Variables	Preintervention	Postintervention	*T*/*Z*	*P* value
Weight (kg)	66.28 ± 10.29	67.22 ± 9.91	-1.348	0.187
BMI (kg/m^2^)	25.03 ± 3.26	25.44 ± 3.43	-1.523	0.138
WC (cm)	87.44 ± 10.45	88.14 ± 11.36	-0.625	0.536
HC (cm)	95.86 ± 5.81	98.27 ± 9.43	-2.035	0.050
SBP (mmHg)	144.00 ± 21.08	145.58 ± 17.08	-0.486	0.631
DBP (mmHg)	82.06 ± 20.79	83.77 ± 12.99	-0.555	0.583
TC (mmol/L)	4.20 ± 1.39	4.14 ± 1.13	0.308	0.760
TG (mmol/L)	1.36 ± 0.90	1.51 ± 1.40	-0.805	0.427
HDL-c (mmol/L)	1.35 ± 0.54	1.34 ± 0.27	0.148	0.884
LDL-c (mmol/L)	2.63 ± 1.11	2.50 ± 0.99	0.875	0.389
FPG (mmol/L)	7.54 ± 2.72	7.20 ± 2.45	0.630	0.533
2hPG (mmol/L)	10.66 ± 3.66	9.30 ± 2.40	2.132	0.041^∗^
HbA1c (%)	7.91 ± 2.16	7.26 ± 1.81	2.722	0.011^∗^
1,5-AG (*μ*g/mL)	96.76 ± 34.87	148.78 ± 31.12	-7.270	<0.001^∗^
CV-FPG (%)	0.19 ± 0.11	0.15 ± 0.12	0.951	0.348
LAGE (mmol/L)	5.84 ± 2.21	3.98 ± 2.32	2.545	0.015^∗^

Data are shown as mean ± SD, ^∗^*P* < 0.05 for baseline vs. after 6-month intervention within-group.

**Table 4:** Comparisons of glycemic control and lipid profile variables within the control group.

Variables	Preintervention	Postintervention	*T*/*Z*	*P* value
Weight (kg)	66.05 ± 11.92	67.02 ± 11.86	-1.962	0.061
BMI (kg/m^2^)	24.45 ± 3.16	24.82 ± 3.21	-2.060	0.050
WC (cm)	87.20 ± 9.56	89.00 ± 10.59	-1.366	0.185
HC (cm)	93.74 ± 6.53	97.50 ± 7.38	-2.871	0.008^∗^
SBP (mmHg)	145.35 ± 18.71	149.87 ± 23.81	-1.247	0.226
DBP (mmHg)	83.74 ± 9.08	85.57 ± 15.76	-0.691	0.497
TC (mmol/L)	4.41 ± 1.03	4.67 ± 0.97	-2.053	0.051
TG (mmol/L)	1.94 ± 1.22	1.63 ± 0.83	1.387	0.178
HDL-c (mmol/L)	1.22 ± 0.41	1.39 ± 0.37	-3.638	0.001^∗^
LDL-c (mmol/L)	2.68 ± 0.99	2.94 ± 0.84	-2.239	0.035^∗^
FPG (mmol/L)	8.48 ± 3.28	7.59 ± 2.82	1.721	0.098
2hPG (mmol/L)	12.25 ± 3.72	12.77 ± 4.21	-0.688	0.499
HbA1c (%)	7.92 ± 2.54	7.31 ± 1.58	1.985	0.059
1,5-AG (*μ*g/mL)	89.33 ± 40.20	110.97 ± 34.41	-2.217	0.037^∗^
CV-FPG (%)	0.20 ± 0.12	0.16 ± 0.88	1.024	0.313
LAGE (mmol/L)	5.96 ± 2.16	6.40 ± 3.08	-0.445	0.662

Data are shown as mean ± SD, ^∗^*P* < 0.05 for baseline vs. after 6-month within-group.

**Table 5:** Between-group differences in the score of the NIHSS and MRS after 6 months.

		Control (*n* = 30)	Intervention (*n* = 33)	*P* value
NIHSS	After	3.50 ± 2.24	2.35 ± 0.81	
	Change	−2.04 ± 1.10	−2.90 ± 1.33	0.012^∗^

MRS	After	1.53 ± 0.90	1.48 ± 0.76	
	Change	−0.80 ± 0.65	−0.91 ± 0.63	0.509

Data are shown as mean ± SD, ^∗^*P* < 0.05. NIHSS: National Institutes of Health Stroke Scale; MRS: modified Rankin scale.

**Table 6:** Comparisons of various dimensions of SIS between groups after 6 months.

Variables	Control (*n* = 30)		Intervention (*n* = 33)	*P* value
Total score	After	559.43 ± 112.53	615.47 ± 87.94	
	Change	59.11 ± 44.73	89.53 ± 70.43	0.141

Strength	After	57.08 ± 19.03	70.83 ± 19.09	
	Change	8.75 ± 15.27	16.67 ± 20.67	0.014^∗^

Memory and thinking	After	78.57 ± 16.86	78.23 ± 18.49	
	Change	−0.71 ± 2.77	0.01 ± 6.59	0.534

Emotions	After	62.04 ± 14.50	62.57 ± 16.35	
	Change	3.89 ± 11.78	8.33 ± 16.32	0.078

Communication	After	90.48 ± 14.58	92.35 ± 13.52	
	Change	7.86 ± 14.98	4.25 ± 9.08	0.068

Activities of daily living	After	79.67 ± 21.79	86.31 ± 15.16	
	Change	12.50 ± 19.18	15.60 ± 20.78	0.716

Mobility	After	81.93 ± 20.91	88.09 ± 15.96	
	Change	11.67 ± 17.58	14.18 ± 20.84	0.387

Hand function	After	53.00 ± 23.13	67.14 ± 18.48	
	Change	2.67 ± 10.33	8.33 ± 14.94	0.016^∗^

Participation	After	56.67 ± 23.38	69.94 ± 15.58	
	Change	12.50 ± 21.68	22.17 ± 19.56	0.171

Data are shown as mean ± SD, ^∗^*P* < 0.05. SIS: Stroke Impact Scale.

**Table 7:** Comparisons of the amount of blood glucose change between groups after 6 months.

Variables		Control (*n* = 30)	Intervention (*n* = 33)	*P* value
FBG (mmol/L)	After	7.54 ± 2.03	6.46 ± 1.00	
	Change	0.90 ± 2.12	−0.38 ± 1.95	0.241
2hBG (mmol/L)				
After breakfast	After	10.10 ± 2.26	9.64 ± 2.72	
	Change	−0.18 ± 0.76	−0.68 ± 2.64	0.692
After lunch	After	10.04 ± 2.82	10.04 ± 3.49	
	Change	−1.02 ± 4.85	−0.64 ± 4.53	0.880
After dinner	After	10.08 ± 2.47	9.69 ± 3.64	
	Change	−1.82 ± 3.92	−0.37 ± 6.64	0.658
Before sleep	After	8.22 ± 1.12	7.68 ± 2.85	
	Change	−2.38 ± 2.94	−0.85 ± 4.13	0.464
Hypoglycemia, *n* (%)	Before	8 (26.7%)	9 (27.3%)	0.958
	After	4 (13.3%)	3 (9.1%)	0.600

Data are shown as *n* (%) and mean ± SD, ^∗^*P* < 0.05. FBG: fasting blood glucose tested by finger stick.

## Data Availability

The data used to support the findings of this study are available from the corresponding author upon request.
